# Modelling & Spatial Mapping of Residential-Sector Emissions for Sub-National & Urban Areas

**DOI:** 10.1016/j.mex.2024.102617

**Published:** 2024-02-16

**Authors:** Lily Purcell, Anna C. O'Regan, Connor McGookin, Marguerite M. Nyhan

**Affiliations:** a School of Engineering & Architecture, University College Cork, Cork, Ireland; b MaREI, the SFI Research Centre for Energy, Climate & Marine, University College Cork, Ringaskiddy, Cork, P43 C573, Ireland; c Environmental Research Institute, University College Cork, Lee Rd, Sunday's Well, Cork, T23 XE10, Ireland; d Delta *E*+ Research Group, Sustainable Energy Engineering, Simon Fraser University, Canada

**Keywords:** Emissions modelling, Emissions inventory, Residential sector, Building emissions, Energy demand, Residential Sector Housing Stock Energy Model & Emissions Inventory

## Abstract

The residential sector accounts for 33% of energy-related Greenhouse Gas (GHG) emissions globally and must undergo rapid emissions reductions in order to support broader society-wide sustainability and net-zero transitions. Additionally, urban areas account for approximately 70% of global GHG emissions. To provide a baseline for urban climate action plans and mitigation strategies, sub-national municipalities must quantify their sectoral baseline emissions in detail and develop strategies for reducing emissions relative to these baselines**.** Therefore, it is important to establish clear methodologies for computing these baselines in accordance with the best available science. This paper establishes a novel methodology for developing a residential sector emissions model using a data-driven and spatial mapping approach. This would form an important component of future multi-sectoral baseline emissions inventories.

• The residential sector emissions model combines publicly available census and building energy performance datasets in order to model and visualize the distribution of energy demand and resultant emissions across an urban study domain in Ireland.
• The methodology presented was developed in line with the approaches and requirements of the Global Covenant of Mayors and the Intergovernmental Panel on Climate Change.
• It is envisioned that this residential sector emissions model methodology could be applied in any urban area worldwide.

## Graphical abstract

**Figure:**
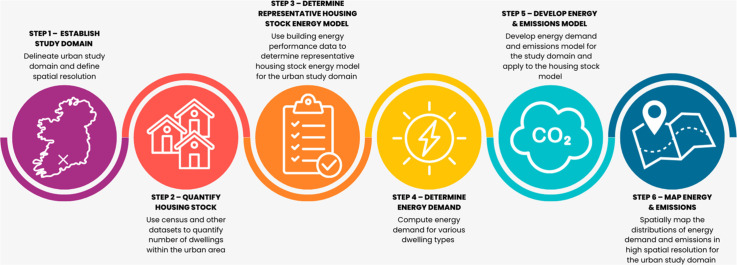

Specifications table

**Table:** 

Subject area:	Engineering
More specific subject area:	Local authority administrative area CO_2_ emissions modelling and mapping
Name of your method:	Residential Sector Housing Stock Energy Model & Emissions Inventory
Name and reference of original method:	• Intergovernmental Panel on Climate Change: 2006 IPCC Guidelines for National Greenhouse Gas Inventories [1]. • Global Covenant of Mayors for Climate and Energy: Common Reporting Framework [2]. • Department of the Environment, Climate and Communications: Local Authority Climate Action Plan Guidelines Technical Annex C [3]. • CODEMA: Developing CO_2_ Baselines: A Step-By-Step Guide for Your Local Authority: [4].
Resource availability:	• Census and New Dwelling Completion datasets available at https://www.cso.ie/en/databases/ • Building energy performance data available at https://ndber.seai.ie • Boundary shapefiles available at https://data.gov.ie/ • Software for statistical analysis and emissions modelling included Microsoft Excel, QGIS, Python and R.

## Background

Mitigating climate change through emissions reductions is an urgent challenge globally, not least due to the devastating impacts that accelerating climate change-associated extreme weather events are having on the environment, infrastructure, food security, economic sectors, society and indeed on public health worldwide [[5], [6], [7], [8]]. There is widespread agreement and acknowledgement that cities and city authorities will play a vital role in the mitigation of climate change and associated temperature increases [[9], [10], [11], [12]]. Currently, the average global temperature is at 1.15°C above pre-industrial levels and recent Intergovernmental Panel on Climate Change (IPCC) reports state that cities account for over 70% of GHG emissions [[6], [8], [13]]. Urbanisation is also occurring at a rapid rate, and 55% of our global population currently resides in urban areas [14,15]. This is expected to increase to 68% by the year 2050 [15]. City authority energy and climate action planning is becoming increasingly important and as such, it is necessary to engage, inform and equip cities in climate mitigation and adaptation efforts [[11], [12],^16^]. The Global Covenant of Mayors for Climate and Energy (GCoM) which began as the European Union's (EU's) Covenant of Mayors and later merged with the Compact of Mayors, is now a global network of 11,500 cities who have committed to climate policies and action including emissions reductions consistent with the Paris Accord's aims of reaching net zero by 2050 and limiting global temperature increases to 1.5 °C above pre-industrial levels within this century [17,18]. Another major city-focused initiative, the EU Mission for Climate-Neutral and Smart Cities, was introduced in 2021. Its goal is to achieve 100 climate-neutral cities by 2030, which would act as exemplars of best practice for all European cities to achieve climate neutrality by 2050 [19]. These global decarbonisation initiatives support the achievement of the United Nations Sustainable Development Goals (SDGs), especially SDG 11 on sustainable cities and communities and SDG 13 on climate action [20], while also acknowledging the importance of advancing and utilising SDG-oriented science, research and innovation in order to ensure progress [21].

One requirement for city authorities joining the GCoM is to develop and submit a Baseline Emissions Inventory (BEI) as a pre-requisite for the creation of a Sustainable Energy and Climate Action Plan [2]. BEIs provide multi-sectoral energy demand profiles and their associated GHG emissions which are a fundamental step in developing sub-national climate action strategies [10]. While this requirement provides a positive precedent, detailed guidelines on the methodological development of BEIs have yet to be provided. They currently only provide sparse recommendations of which sectors to include and a suggestion for an activity-based approach over a life-cycle assessment approach [9]. Activity-based approaches refer to emissions in source categories or sectors within a geographical area or community, usually over a yearly period, whereas life-cycle assessments evaluate specific products from raw material extraction to the end of life of the product [[9], [22]]. The IPCC provide slightly more in-depth guidance and provides potential sources of data while also advising authorities to follow one of three ‘tiered’ approaches. These range from a Tier 1 approach which consists of simple calculation methods and default emissions factors to a Tier 3 approach which applies models and country-specific emissions factors. However, these guidelines were developed for the estimation of emissions inventories at a national level only and do not provide detailed methodologies for developing local or city-wide BEIs [1]. As such, there is an urgent need for clear guidelines on the methodological development of multi-sectoral BEIs at a sub-national and urban level.

In response to this knowledge gap, this paper outlines a methodology for residential sector emissions modelling, in line with the recommended GCoM ‘activity-based’ approach and IPCC Tier 3 BEI approach. This covers electricity for appliances and lighting, as well as both domestic space and water heating, which is also referred to as ‘stationary energy’ by the IPCC and the GCoM [[1], [2],^6^]. Additionally, the methodology describes a spatial mapping approach which visualizes the distribution of energy demand and CO_2_ emissions. Benefits of this approach include informing policy, for example, spatial mapping can inform area-based approaches to retrofitting, building energy improvements and more targeted transport infrastructure developments. Spatial mapping may also assist in raising public awareness of emissions within the urban area of interest. Although spatial modelling and mapping is used widely in the portrayal of urban environmental metrics including air pollution [[23], [24], [25]], urban greenspace [26] and transportation emissions [27,28], it is not widely applied in modelling emissions from the residential sector [29]. In some studies where spatial mapping has been applied, method details or data collection are limited [30], emissions have not been disaggregated by sector [31] or not all sources of emissions have been included [32] making these unsuitable for all-encompassing multi-sector BEIs. As such, we respond to that requirement specifically in the context of the residential sector.

Previous research on residential sector emissions has focused mainly on analyzing building efficiency [[33], [34], [35]] as opposed to residential sector BEIs. In an Irish context, for the purpose of emissions reporting, residential sector emissions only include the combustion of fuel for domestic space and water heating, while electricity is accounted for upstream at the power plant under ‘energy industries’ [36]. However, attributing the emissions to demand (i.e. residential lighting or appliances) is more useful for energy planning and identifying mitigation measures such as more energy efficient lighting or demand reductions.

Countries globally are developing robust national strategies for emissions reductions. For example, Ireland developed a national Climate Action Plan, detailing cross-sectoral emissions reduction targets. Furthermore, the Irish Climate Amendment Act 2021 mandates each municipality to develop sub-national five-year climate action plans. They are required to include a BEI along with emissions mitigation and climate adaptation measures for their administrative areas. Sub-national BEIs and accompanying strategies enable municipalities and their citizens worldwide to identify emissions-intensive activities and develop sectoral pathways for achieving local emissions reduction targets which in turn contribute to emissions mitigation nationally. However, approaches for BEIs are not well established at the sub-national, urban or local level. Working at a sub-national level is challenging due to data not being available in sufficiently high spatial resolution for developing local-level building stock energy and emissions models [37,38]. With the exception of Sweden and Denmark, where sub-national and local energy planning is well-established, there is a lack of transparency and consistency in the approaches employed (McGookin et al., 2021). As such, it is important to establish clear methodologies for computing these emissions baselines using the best available science, and clear methodologies for spatially modelling and mapping residential-sector emissions for sub-national and urban areas.

## Datasets

The methodology for modelling and spatially mapping residential-sector emissions was developed to support the establishment of a BEI for an urban area in Ireland. Two main data sources, namely the Central Statistics Office (CSO) and the Sustainable Energy Authority of Ireland (SEAI), were employed for the study domain of Cork City. Please see Table 1 for a full list of data sources used. The CSO collect, process and publish data about Ireland and its citizens and is responsible for the National Census that takes place every five years. The smallest spatial unit used by the CSO is the Census Small Area (SA) level. SAs are statistical boundaries containing approximately 80-120 households identified by their unique SA code. Therefore, the emissions inventories and maps in this study were computed for this spatial resolution using existing census data. These data were merged with building energy datasets to subsequently infer trends about the distribution of energy use and emissions across the city. In Ireland, local authority climate action plan guidelines advise emissions inventories to be quantified for the baseline year of 2018. The 2016 Census Small Area Population Statistics included the most relevant data available as it contained information on the Irish housing stock for the year 2016. Additionally, the CSO publishes New Dwelling Completions (NDCs) every three months and to supplement the census dataset, NDCs for 2017 and 2018 were acquired [39,40].

**Table 1:** Sources of data used in the residential sector BEI method for an urban study domain in Ireland.

Resource/Dataset	Data source
Energy performance and characteristics of dwellings	BER Research Tool [48]
Number of dwellings and types per Small Area	2016 Census [39]
Newly built dwellings completed since 2016 Census	2017 & 2018 New Dwelling Completions [40]
Emission conversion factors	Conversion Factors [48]; Energy in Ireland 2019 Report [49]
GIS shapefiles	Study domain boundary [43]; Small Area statistical boundaries [44]

The SEAI is an Irish governmental organization responsible for energy reporting, distributing grants (e.g. solar PV or EV support) and information of sustainable energy or energy improvement measures. They also manage the Building Energy Rating (BER) database, which grades the energy performance of homes on a scale from A (most efficient) to G (least efficient). The BER is equivalent to an Energy Performance Certificate (EPC) and is commonly used across the EU [41]. Since January 2009, all households in Ireland which are for sale or for rent are required to obtain a BER Certificate by means of assessment. The SEAI's BER Research Tool is a public database containing all anonymized BER assessments to date and provides insights into home energy demand. Details include typical space heating requirements, water heating requirements, lighting, and fuel consumption, and details on household type, sizing and energy efficiency. The BER database provides further detail on household type than the CSO datasets, categorizing households across eleven household type categories. Assessments within the BER database also include each household's Census SA code [42].

To quantify and map the distribution of energy demand and associated GHG emissions in high spatial resolution across the city, publicly available Geographical Information System (GIS) shapefiles were used. These included a shapefile containing all SAs in Ireland and the Cork City municipality boundary shapefile which was used to isolate the SAs which are located within the urban case study of interest [[43], [44]].

## Methods

### Step 1 – Establish Urban Study Domain Boundary

BEIs must be prepared for an appropriate baseline year in order to compare with preceding reports, track progress over time and inform strategies in the future. For this reason, the extension of the urban study area boundary in 2019 was factored into the methodology despite occurring after the baseline year of 2018. This was completed in order to contribute necessary foresight regarding future reports. Consequently, census datasets organized by ‘County and City’ were inapplicable in this context. However, as stated previously, a finer spatial resolution was requested with this BEI approach and the census dataset acquired and described previously provides data on households at the SA level. Thus, a CSV file was downloaded from the source website and imported into a GIS software system, namely QGIS version 3.28.1, before being ‘joined’ to a shapefile of all SAs nationally [45]. An updated boundary file of the urban study domain was overlaid with this. In order to isolate data for just the SAs within the study domain, the ‘clip’ tool was used to extract the relevant data. 856 SAs were entirely or partially within the new city boundary. From this, 13 were excluded from the analysis since just 10% of their total area was within the domain boundary. This resulted in 843 SAs for analysis. For the remaining SAs, it was assumed that the proportion of land within the boundary was representative of the proportion of dwellings to be included in the housing stock of the study domain. For example, if 20% of the area was inside the boundary then 20% of the houses were included.

### Step 2 – Quantify Housing Stock

The census data extracted for 843 SAs was then processed to compute the total number of dwellings in the city up to the census’ year of completion which was 2016. The number of new dwellings constructed in 2017 and 2018 were then added to these to obtain the total number of dwellings in each SA and across the city (*n* = 78,856) at the end of the baseline year.

### Step 3 – Determine Representative Housing Stock Energy Model From Building Energy Performance Data

An urban housing stock energy model was then developed using the BER dataset. Firstly, the raw data was imported into QGIS and data entries with SA codes located within the study domain were extracted. As the assessment entries are compiled and updated over time, assessments dated after the baseline year were removed. Using a similar method to Step 1, a proportional number of entries were included for SAs bisected by the domain boundary.

This refined BER assessment dataset (*n* = 40,121) included eleven dwelling type categories including ‘apartment’, ‘basement dwelling’, ‘detached house’, ‘end of terrace house’, ‘ground-floor apartment’, ‘house’, ‘maisonette’, mid-floor apartment’, ‘mid-terrace house’, ‘semi-detached house’, and ‘top-floor apartment’. These were aggregated into the four categories of ‘detached’, ‘semi-detached’, ‘mid-terrace’ and ‘apartment’. Maintaining the split into these four categories is important to ensure that the different size of dwellings (i.e. floor area) is taken into account. BER ratings were also aggregated by the letters A to G. A full list of the dwelling type groups and BER sub-categories can be found in the Supplementary Material (see Figs. S1 and S2). The BER entries were then grouped by dwelling type and energy rating in order to form a representative profile of the housing stock for the study domain (see Table 2 for details).

**Table 2:** Profile of Building Energy Rating (BER) dwelling entries categorized by dwelling type and BER sub-group as well as dwelling-type totals (*N* = 40,121). Representative housing stock including numbers of dwellings and percentage share (%) per sub-group are shown. Percentages may not total 100 due to rounding.

### Step 4 - Determine Energy Demand

The BER dataset provides energy demand values for numerous services including lighting, pumps and fans, main space heating, secondary space heating, main water heating and supplementary water heating. After the BER data entries were assigned to dwelling-type categories, they were isolated and analysed by BER rating sub-group, A-G. Within each BER rating sub-group, the mean energy demand for all services were computed in each dwelling-type category (see Table 3 for an example for the ‘detached house’ category). The building energy rating is based on building fabric and heating system omitting electrical demand except for lighting, pumps and fans. This missing data was addressed by adding appliance and cooking loads based on the national average energy demand in households [46]. Lighting and appliances generally account for 17% of energy demand. Energy demand from appliances was modelled using existing delivered lighting energy values and the newly calculated lighting and appliances demand. Appliance loads were then added to the existing delivered energy from lighting, pumps and fans in order to obtain the electrical demand loads. The loads from electrical demand and cooking can be seen in the last two columns of Table 3.

**Table 3:** Sample of energy demand sources for detached households, their associated mean energy delivered for main space heating and secondary space heating (kWh/m^2^/yr) and their corresponding mean total floor area (m^2^) across the Building Energy Rating (BER) sub-groups A-G. Similar data was generated for all other sources of energy demand for each dwelling-type category.

The overall energy demand from all services was computed for each BER sub-group in all dwelling-type categories. This was then multiplied by the sub-group's corresponding number of BER entries in Table 2, resulting in a sub-group total energy demand within each dwelling-type category. By summing all BER sub-group energy demand totals and dividing by the number of entries within each dwelling-type category, the weighted mean annual energy demand for each dwelling type was determined using Eq. (1) as follows:

(1) $$\begin{array}{c} ED_{i}\;=\;\frac{\sum_{n=1}^{j}\left(d_{\mathrm{ij}}\;x\;n_{\mathrm{ij}}\right)}{n_{i}} \end{array}$$

where, ED_i_ is the weighted mean annual energy demand per dwelling-type category, *i*, per annum; *d_ij_* is the BER sub-group energy demand totals for each dwelling-type category, *i*, and BER sub-group, *j*, (A-G) per annum; *n_ij_* is the number of dwellings in the BER sub-group, *j*, of the dwelling-type category, *i*; and, *n_i_* is the number of dwellings in the dwelling-type category, *i*. The weighted mean was computed as some BER sub-groups had more dwellings than others and some building energy ratings, A-G, were more prevalent in the data.

### Step 5 – Develop Residential Sector Energy & Emissions Model

Step 5 brings together the results of the previous steps; energy demand (Step 4) and housing stock profile (Steps 2 & 3). There were two issues that needed to be addressed in order to obtain the desired results for each SA. Firstly, not all homes have a BER. On average, 30% of homes across the SAs had a BER, but there were also some SAs with no BERs. Secondly, the Census and NDC datasets did not include the number of homes per dwelling types (i.e. semi-detached, apartment, etc.) in any individual SA within the urban study domain. To address this, the BER shares presented in Table 2 were applied to the total number of homes.

A model was developed using Python version 3.8.16 [47] to identify the number of each dwelling type in every SA and in the study domain overall. Following the identification of the number of each dwelling type, the housing stock, quantified in Step 2, was multiplied by the share of each dwelling type in every SA. For SAs with no BER data (*n* = 2), the overall profile of dwelling types across the city was assigned. The mean energy demand for the four dwelling types (kWh/yr by BER rating) which was computed in Step 4, was then multiplied by the number of each dwelling type in every SA and aggregated to determine SA sub-totals as well as the total energy demand for the overall study domain.

Subsequently, the entries in the BER assessment dataset were grouped by fuel use for main space, secondary space and water heating, in order to determine a fuel-share profile for each dwelling-type category (see Table 4). The total dwelling-type energy demand computed previously was then multiplied by the dwelling-type's fuel-share profile to quantify energy demand by fuel type. Energy to CO_2_ conversion factors (in gCO_2_/kWh) were obtained for the baseline year [[48], [49]] and applied to compute the total annual emissions for each dwelling type. This was computed using Eq. (2) as follows:

(2) $$RE_{i}\;=\;\sum_{n=1}^{k}\left(RED_{ik}\;\times \;EF_{k}\right)$$

where, *RE_i_* is the residential emissions per dwelling-type category, *i*, per annum; *RED_ij_* is the residential energy demand for each dwelling-type category, *i*, and fuel-type, *k*, (natural gas, heating oil, liquid propane gas [LPG], electricity and solid multi-fuel) per annum; and, *EF_k_* is the emissions factor for each fuel-type, *k*.

**Table 4:** Overall fuel share profile including numbers (and corresponding percentage) of dwellings perdwelling type and fuel category. Percentages sum up to 100% for each dwelling type (row).

Dwelling Type	Natural Gas	Heating Oil	LPG	Electricity	Solid Multi-Fuel
*Detached*	8,662 (50.6%)	3,230 (18.9%)	143 (0.8%)	974 (5.7%)	4,098 (24.0%)
*Semi-detached*	28,839 (60.0%)	4,539 (9.4%)	54 (0.1%)	2,855 (5.9%)	11,800 (24.5%)
*Mid-terrace*	18,110 (60.1%)	1,692 (5.6%)	63 (0.2%)	2,976 (9.9%)	7,278 (24.2%)
*Apartment*	6,993 (38.9%)	88 (0.5%)	16 (0.1%)	9,912 (55.1%)	974 (5.4%)

Finally, the total annual dwelling-type emissions was divided by the number of dwellings within all dwelling-type categories to determine mean annual CO_2_ emissions per household. The mean CO_2_ for each dwelling-type was then multiplied by the total number of each dwelling type in the housing stock. These were summed to compute total residential sector emissions for each SA and the study domain.

### Step 6 - Spatially Map Residential Sector Energy Demand and Emissions

**Fig. 1:**
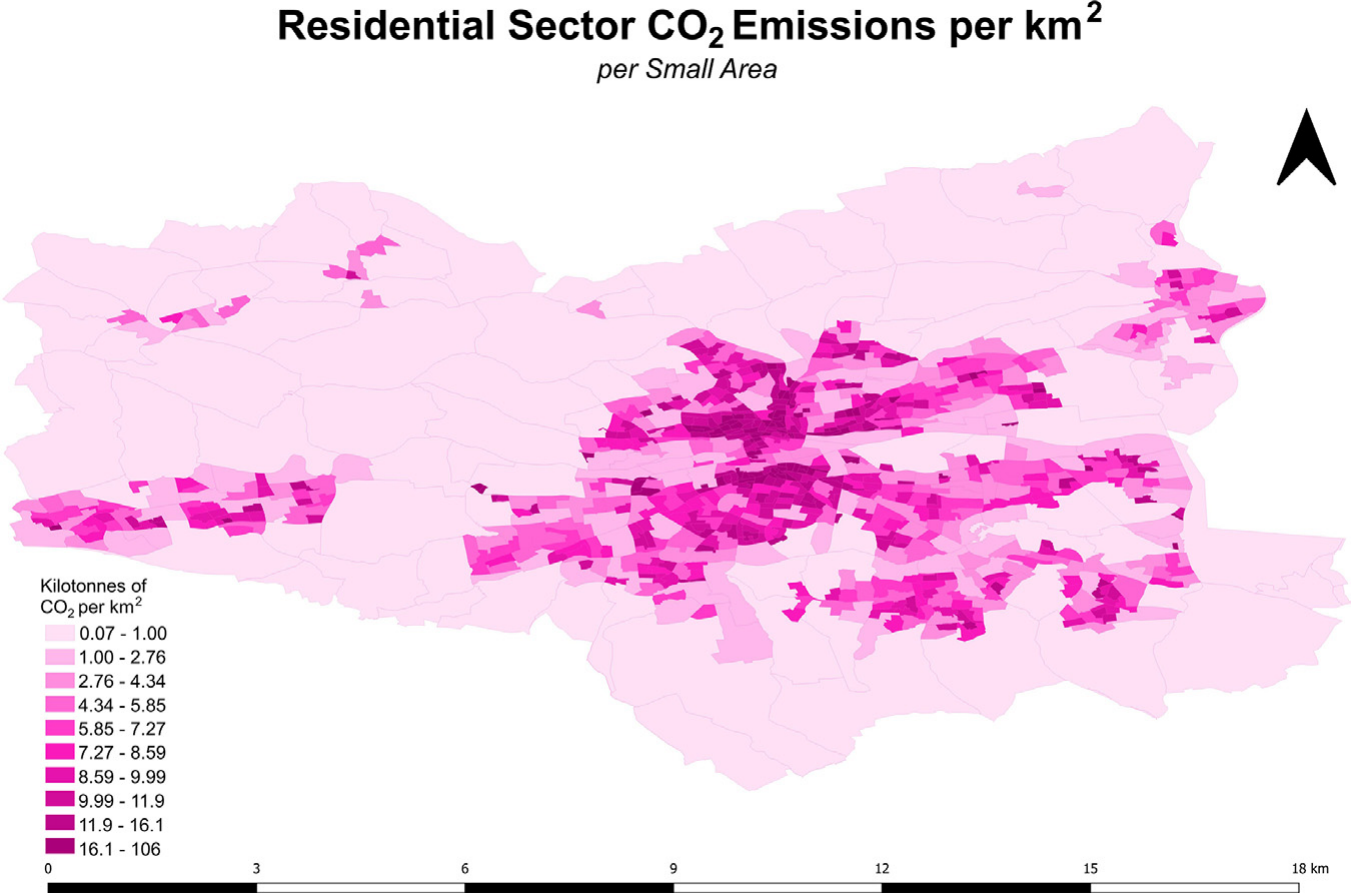
Spatially mapped CO_2_ emissions density for the residential sector for all Small Areas within the Cork City administrative boundary. Units are kilotonnes of CO_2_ per square kilometer (ktCO_2_/km^2^).

It was essential to model the total annual energy demand and emissions for all 843 SAs in order to map energy demand and emissions in high spatial resolution across the study domain. In order to account for the variability of SA sizes and standardize absolute CO_2_ emissions across the study domain, emissions densities including CO_2_ per km^2^, CO_2_ per dwelling and CO_2_ per person were also derived and mapped for each SA. This was completed by importing a CSV file of the computed energy and emissions and their associated SA identifiers into QGIS and ‘joining’ these to the shapefiles imported previously in Step 1. A map of the modelled CO_2_ emissions per km^2^ per SA can be seen in Fig. 1 while additional energy and emissions maps for the study domain can be seen in the Supplementary Material (Figs. S3–S7). As BEIs and accompanying maps are to assist municipalities in developing decarbonizing strategies, the density of energy demand from heat only, in GWh per km^2^, within each SA was also computed and mapped in order to highlight potential locations within the city which would be suitable for district heating. This map can also be found in the Supplementary Material (Fig. S5).

## Conclusion

This paper presents detailed guidelines for developing a baseline emissions inventory for the residential buildings sector. There is an increasing demand for local governments and city authorities to develop multi-sector climate action plans including emissions reductions targets. This is of particular concern for the residential buildings sector which exhibits high energy demand and subsequent emissions. Effective mitigation strategies must include the completion of a baseline emissions inventory. However, to date, specific methods relating to the residential sector have not been established. This paper describes a novel method for modelling and spatially mapping residential-sector emissions and is applicable in any urban context where census and building energy performance data are available. The resulting energy, emissions and map outputs can be used by municipalities who are developing decarbonization strategies for the energy-intensive residential sector. They may also form part of an all-sector BEI, which is a vital step in the development of municipal climate action plans and the transition towards net-zero emissions in urban areas. It is recommended that future research on emissions inventories should consider developing and publishing detailed methods for other sectors in an effort to further support sub-national and urban emissions mitigation efforts.

## Supplementary Material

Supplementary material associated with this article includes a table of the available statistical spatial resolutions in the Republic of Ireland and additional maps of the distribution of energy and emissions metrics across the urban study domain.

## CRediT authorship contribution statement

**Lily Purcell:** Conceptualization, Formal analysis, Methodology, Software, Visualization, Writing – original draft. **Anna C. O'Regan:** Data curation, Formal analysis, Software, Visualization. **Connor McGookin:** Conceptualization, Data curation, Methodology, Validation, Writing – review & editing. **Marguerite M. Nyhan:** Conceptualization, Methodology, Supervision, Writing – original draft, Writing – review & editing, Funding acquisition.

## Declaration of competing interest

The authors declare that they have no known competing financial interests or personal relationships that could have appeared to influence the work reported in this paper.

## Data Availability

The links to all datasets have been included in the Specifications Table and in the Reference List

## Appendix Supplementary Materials

Image, application 1
