# Supplementary material for: Modelling & Spatial Mapping of Residential-Sector Emissions for Sub-National & Urban Areas
Source: MethodsX. 2024 Feb 16;12:102617. doi: 10.1016/j.mex.2024.102617 (PMC10901902; doi:10.1016/j.mex.2024.102617)
Supplement: Supplementary file 1 [file mmc1.docx]

**Supplemental Material for:**

Modelling & Spatial Mapping of Residential-Sector Emissions for Sub-National & Urban Areas

Lily Purcell^a,b,c,*^, Anna C. O’Regan^a,b,c^, Connor McGookin^a,b,c,d^, Marguerite M. Nyhan^a,b,c^

*^a^School of Engineering & Architecture, University College Cork, Cork, Ireland.*

*^b^MaREI, the SFI Research Centre for Energy, Climate & Marine, University College Cork, Ringaskiddy, Cork, P43 C573, Ireland.*

*^c^Environmental Research Institute, University College Cork, Lee Rd, Sunday’s Well, Cork, T23 XE10, Ireland*

*^d^Delta E+ Research Group, Sustainable Energy Engineering, Simon Fraser University, Canada*

Corresponding author: L. Purcell. Email: [*118409576@umail.ucc.ie*](mailto:*118409576@umail.ucc.ie)


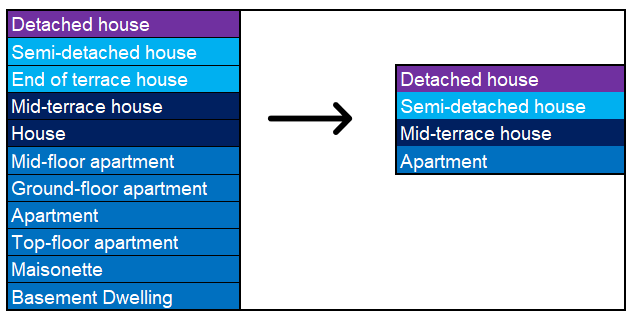


Figure S1. Previous dwelling-type categories listed in the Building Energy Rating (BER) assessment dataset and new dwelling-type categories employed in the residential sector energy and emissions model. Dwelling types listed are colour-formatted in accordance with the new category they were assigned to.

| **Building Energy Rating** | **Combined BER groups** |
| --- | --- |
| A1 | A |
| A2 |  |
| A3 |  |
| B1 | B |
| B2 |  |
| B3 |  |
| C1 | C |
| C2 |  |
| C3 |  |
| D1 | D |
| D2 |  |
| E1 | E |
| E2 |  |
| F | F |
| G | G |

Figure S2. Building Energy Rating (BER) assessment ratings were aggregated into seven rating groups from A-G.


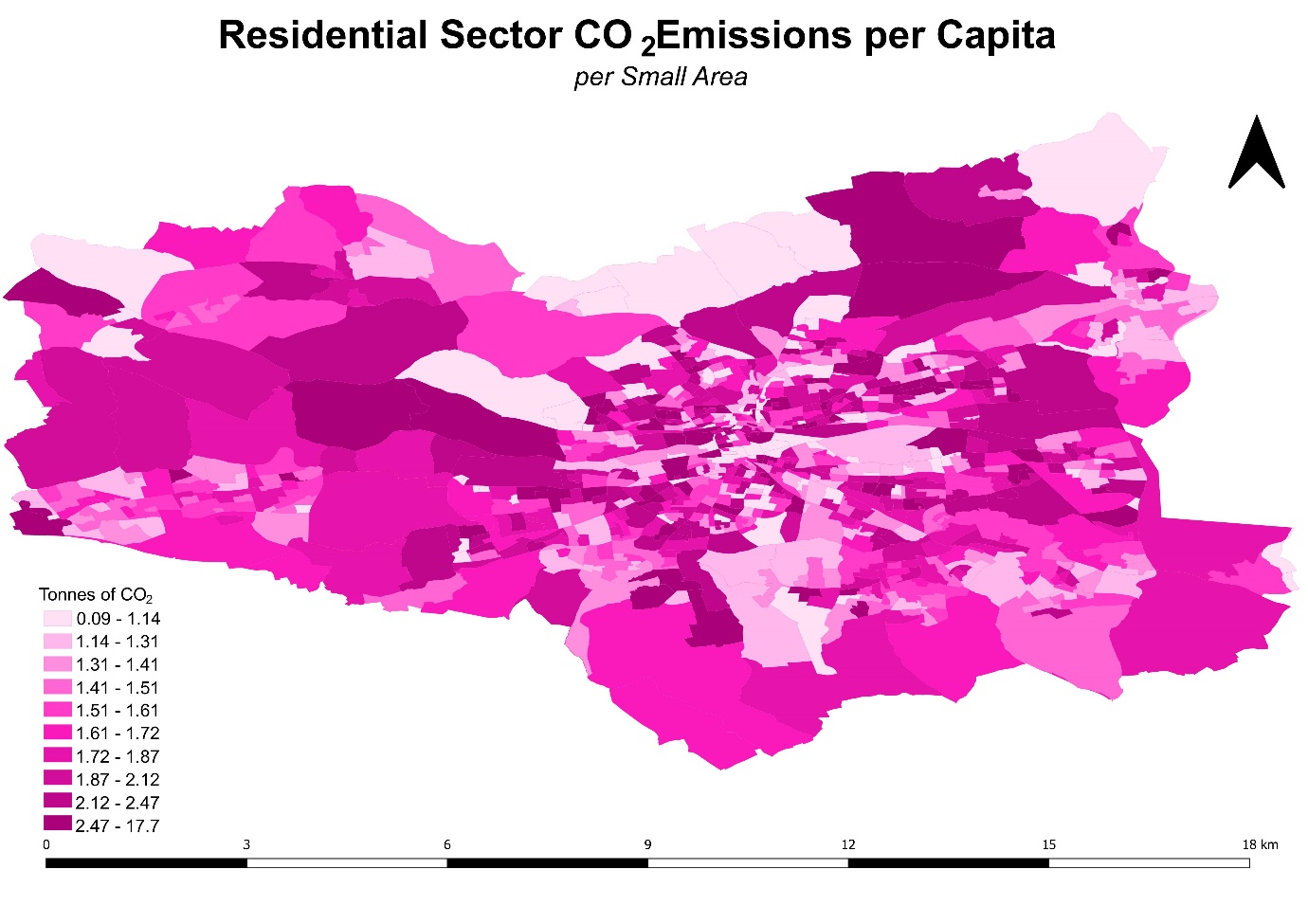


Figure S3. Spatially mapped CO_2_ emissions density for the residential sector for all Small Areas within the Cork City administrative boundary. Units are tonnes of CO_2_ per capita (tCO_2_/capita).


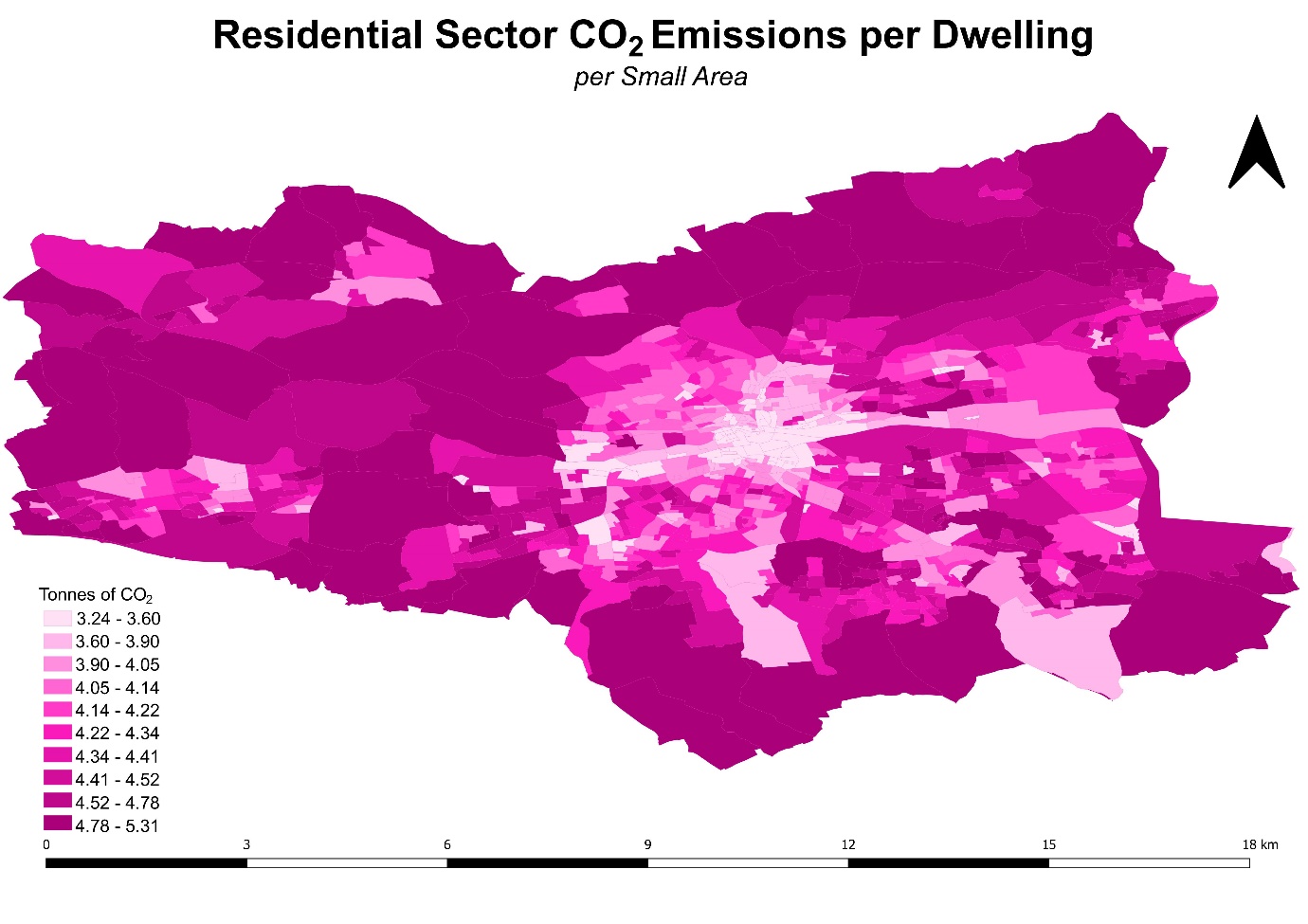


Figure S4. Spatially mapped CO_2_ emissions density for the residential sector for all Small Areas within the Cork City administrative boundary. Units are tonnes of CO_2_ per dwelling (tCO_2_/dwelling).


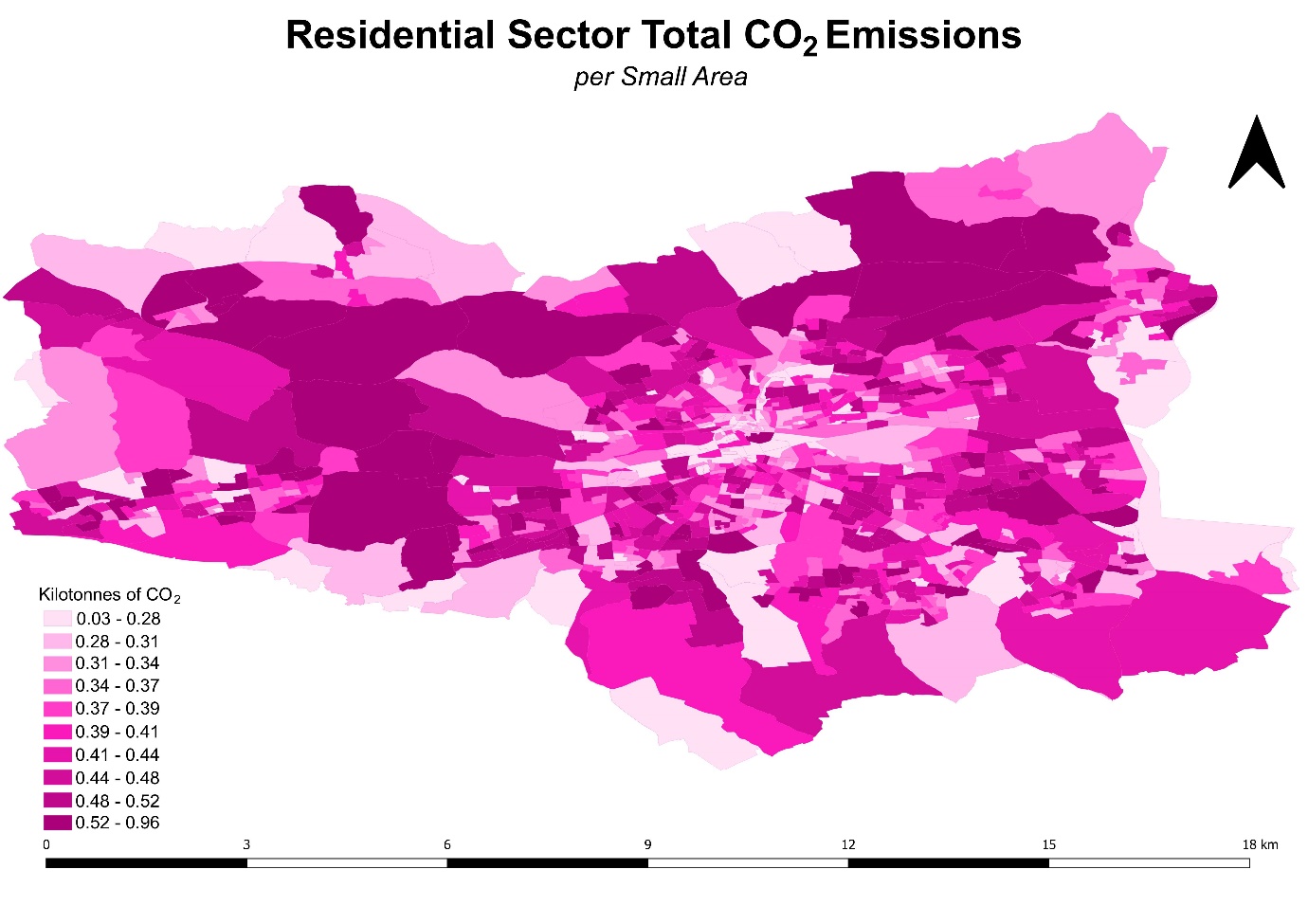


Figure S6. Spatially mapped total CO_2_ emissions for the residential sector for all Small Areas within the Cork City administrative boundary. Units are kilotonnes of CO_2_ (ktCO_2_).


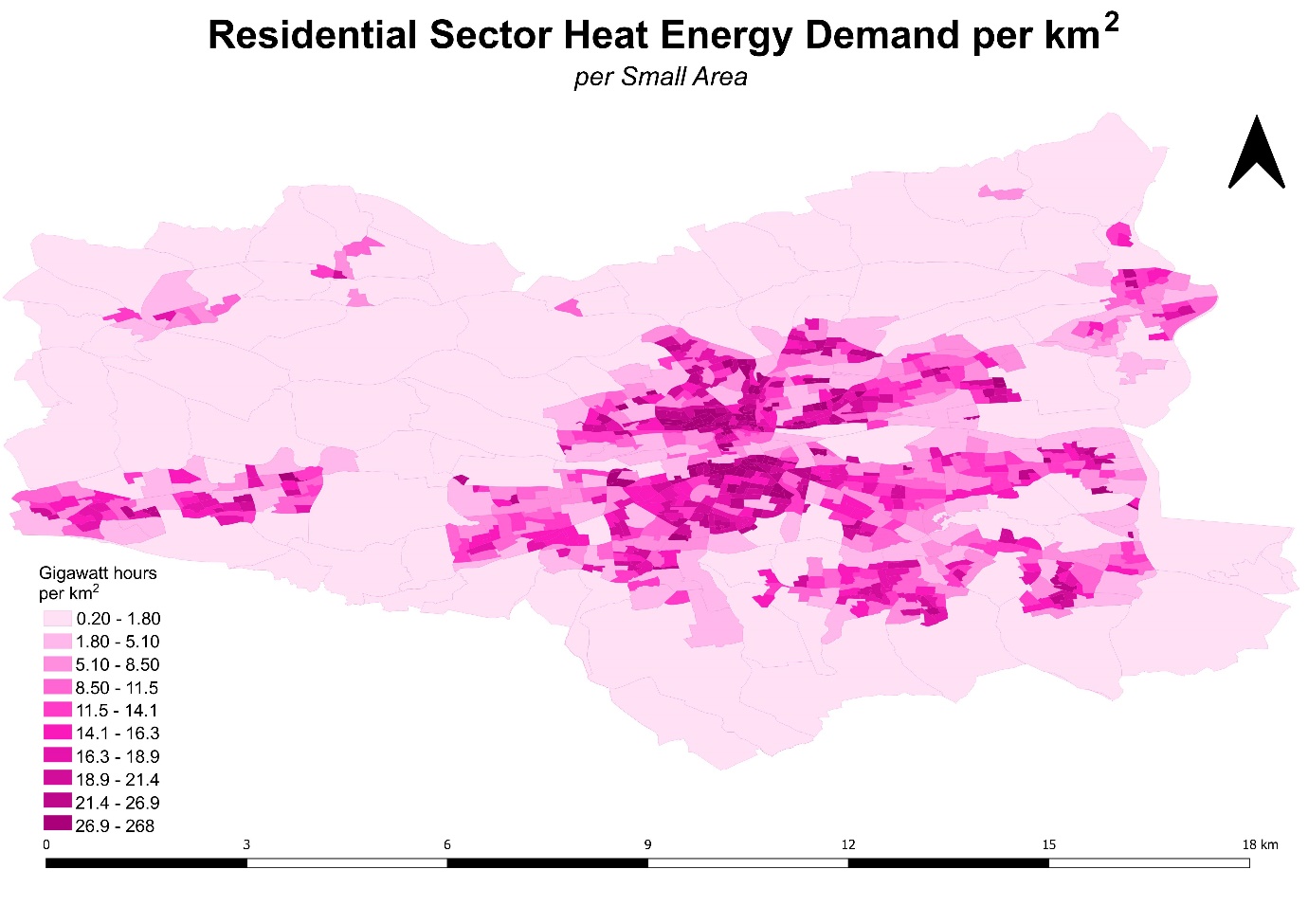


Figure S5. Spatially mapped heat energy demand density for the residential sector for all Small Areas within the Cork City administrative boundary. Units are gigawatt hours per square kilometer (GWh/km^2^).


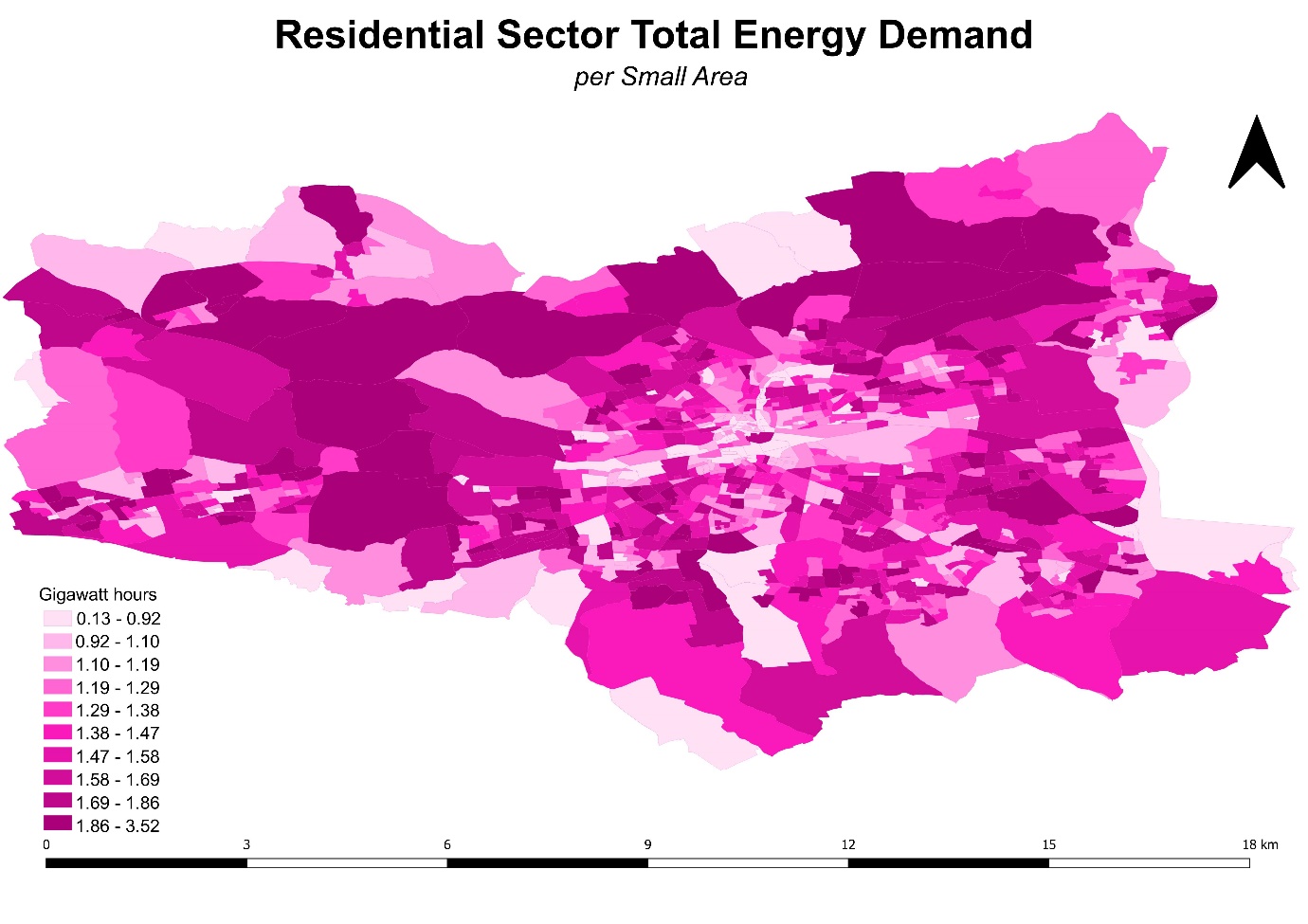


Figure S7. Spatially mapped total energy demand for the residential sector for all Small Areas within the Cork City administrative boundary. Units are gigawatt hours (GWh).
